# Editorial: Radiation as Risk Factor, Early Diagnosis, Therapy, and Follow-up of Differentiated Thyroid Cancer

**DOI:** 10.3389/fendo.2021.797969

**Published:** 2021-12-06

**Authors:** Christoph Reiners, Valentina Drozd

**Affiliations:** ^1^ University Hospital Würzburg, Würzburg, Germany; ^2^ Department of Nuclear Medicine, Würzburg, Germany; ^3^ Foundation Arnica, Minsk, Belarus

**Keywords:** differentiated thyroid cancer, diagnosis, treatment, follow-up, treatment side effects

## Introduction

There are a number of risk factors for differentiated thyroid cancer (DTC) that have been well known for many years (1, 2). Among them are the female gender (3), a family history of thyroid cancer, and some inherited diseases, e.g., familial adenomatous polyposis and Cowden disease (4).

The most relevant risk factor for DTC without any doubt is radiation exposure of the thyroid in childhood (5). There is some evidence that follicular thyroid cancers are more prevalent in iodine deficient populations and that benign thyroid diseases (like goiter) or obesity are risk factors for DTC too (6, 7). Recently, environmental pollutants like nitrate have been blamed for increasing the risk of DTC with combined radiation exposure to the thyroid gland (8).

Unfortunately—with the exception of one paper on radiation exposure and another one on genetic predisposition—no articles were submitted to better understand these risk factors and to develop strategies for prevention of DTC in a broad sense. Nevertheless, the 12 papers of this Research Topic focusing on diagnosis, treatment, and follow-up of DTC present interesting information relevant for patient care.

## Radiation Exposure/Genetic Predisposition

Drozdovitch from Bethesda, USA contribute a review on “Radiation Exposure to the Thyroid after the Chernobyl Accident” presenting individual and population-average thyroid doses after Chernobyl. Mean individual doses in screening cohorts amount to 0.27 Gy in Belarus and 0.19 Gy Ukraine. In evacuees of Belarus, 0.68 Gy are reported for adults and 3.1 Gy for children of 0-7 years old, whereas these doses for Ukrainian evacuees amount to 0.28 Gy in adults and 1.2 Gy in children. Without any doubt, theses doses in children are relevant for DTC induction. Importantly, more than 90% of theses doses in children could have been prevented if milk and food contaminated with I-131 would have been withdrawn.

Mussazhanova et al. from Nagasaki, Japan investigate “The Contribution of Genetic Variants to the Risk of Papillary Thyroid Carcinoma in the Kazakh Population: Study of Common SNPs and their Clinicopathological Correlations”. Single nucleotide polymorphisms (SNIPs) were genotyped in a cohort of 485 PTC patients and a control group of this population (N=1,008), both not exposed to radiation. Five SNIPs adjusted for age and sex show a suggestive association with unidirectional independent effects of FOXE1/PTCSC2, FOXE1 5’UTR, NRG1 intron1, PTCSC3/NKX2-1, BATF upstream contributing to 30-40% of PTC risk.

## Diagnosis

Shi et al. from Beijing, China review the “Diagnostic Value of Sonographic Features in Distinguishing Malignant Partially Cystic Thyroid Nodules” performing a meta-Analysis of 8 high quality studies with 2,004 patients. The highest odds ratios (OR) are described for eccentric localization (OR 17.22 (6.53–45.41), marked or mild hypo-echogenecity [OR 5.97 (2.47–14.43)], and microcalcification [OR 38.76 (6.10–31.97)].

Wu et al. from Taipei, Taiwan investigate human observers versus computer software comparing US TIRADS criteria versus FNA Bethesda malignancy risk categories “Risk stratification in patients with follicular neoplasm on cytology: use of quantitative characteristics and sonographic patterns”. Interestingly, the computer system leads to no significant diagnostic improvement. However, the patient sample of 22 DTC cases compared to 43 benign nodules is too small to derive definitive conclusions.

In a small patient sample too, Peng et al. from Changsha, China report on “Petal-like calcifications in thyroid nodules on ultrasonography: a rare morphologic characteristic of calcification associated with aggressive biological behavior”. The authors describe flower-petal like calcifications defined as many hyperechogenic spots around solid nodules in 18 cases of PTC, 13 of which had central and 5 of which had lateral lymph node metastasis.

Chen et al. from Changsha, China contribute a case report on “Contrast-enhanced ultrasound of primary squamous cell carcinoma of the thyroid”. In a 59 year old male with clinically aggressive cancer, reduced blood flow is described as a typical sign of squamous cell cancer of the thyroid.

In a single center comparison of 174 PTC and 12 control cases, Zhang et al. from Zhengzou China study “Fine needle biopsy versus core needle biopsy combined with/without thyroglobulin or BRAF 600E mutation assessment for detecting cervical nodal metastasis of papillary thyroid carcinoma”. The authors conclude that sensitivity and specificity of fine needle aspiration biopsy cytology can be increased by combining it with thyroglobulin measurement in the washout. However, 12 control cases are not sufficient for a statistically reliable judgment.

## Treatment/Follow-Up

A comprehensive review on circulating RNAs, Xu and Jing from Taiyuan, China explore how “Advances on circRNAs contribute to carcinogenesis and progression in papillary thyroid carcinoma”. The authors discuss its regulatory roles, pathological mechanisms, and their clinical and therapeutic significance. They show that dysregulated circRNAs correlate with aggressive clinical behavior of PTC. For predicting prognosis more long-term follow-up studies are needed. The role of these biomarkers for targeted therapy is still unclear.

“Predictive value of thyroglobulin changes for the curative effect of radioiodine therapy in patients with metastatic differentiated thyroid carcinoma” is the title of a contribution of Wang et al. from Tianjin, China. In this single center study 117 patients with metastatic DTC, 71% showed partial or complete remission after radioiodine therapy. Mean Delta-TG of 22% on TSH-suppressive levothyroxine treatment predicted response with a sensitivity of 86.7% and specificity of 88.2%.

In a laborious single center study with 202 patients (median follow-up 10.7 years), Barros-Filho et al. from Sao Paulo, Brazil propose that “GADD45B transcript is a prognostic marker in papillary thyroid carcinoma patients treated with total thyroidectomy and radioiodine therapy”. Alterations of BRAF, RAS, RET and TERT were the transcriptome profiled in a subgroup of 48 patients. No mutation was associated with the recurrence risk. However, 8 promising genes were identified showing down-expression in the response group after thyroidectomy and radioiodine therapy exclusively. As the best candidate, GADD45B transcript is described as an independent marker of recurrence with shorter disease-free survival and a hazard ratio of 2.9 (95% 1.2-7.0).

## Treatment/Side Effects

Reiners et al. from Würzburg, Germany contribute a review of the literature and results of a multi-registry survey in 7,565 female DTC patients on “Breast Cancer after Treatment of Differentiated Thyroid Cancer With Radioiodine in Young Females: What We Know, and How to Investigate Open Questions”. The general breast cancer risk in DTC survivors is low, ∼2%, slightly higher in females than in males. RAI presumably does not substantially influence breast cancer risk after DTC. However, data from patients given RAI at young ages are sparse and insuffucient to make definitive conclusions regarding age dependence of the risk of breast cancer as a SPM after RAI of DTC. In conclusion, a potential international multicenter study of female patients undergoing RAI of DTC as children, adolescents, or young adults, with a sufficient sample size, is recommended.

In the same context, Drozd et al. from Minsk, Belarus performed a feasibility study in 111 females from Belarus with DTC after RAI and a control group of 90 DTC patients without RAI. The title of the study is “Feasibility Study Shows Multicenter, Observational Case-Control Study is Practicable to Determine Risk of Secondary Breast Cancer in Females with Differentiated Thyroid Carcinoma Given Radioiodine Therapy in their Childhood or Adolescence; Findings also Suggest Possible Fertility Impairment in Such Patients”. The main objective of the study was to develop and test a comprehensive protocol, which later could be used for a consecutive study in much larger, multicenter international patient sample. The protocol developed is described as comprehensive and practicable.

Additional findings in this small sample are that patients given RAI significantly less frequently need 2nd surgeries than controls (23%, 26/111 vs. 39%, 35/90, P < 0.05) and that RAI patients appear to have more frequent reproductive difficulties than controls: 78% (87/111) of the former vs. 93% (84/90) of the latter had a history of pregnancy (P < 0.001).

## Author Contributions

Both authors listed have made a substantial, direct, and intellectual contribution to the work and approved it for publication.

## Conflict of Interest

The authors declare that the research was conducted in the absence of any commercial or financial relationships that could be construed as a potential conflict of interest.

## Publisher’s Note

All claims expressed in this article are solely those of the authors and do not necessarily represent those of their affiliated organizations, or those of the publisher, the editors and the reviewers. Any product that may be evaluated in this article, or claim that may be made by its manufacturer, is not guaranteed or endorsed by the publisher.
